# Diagnostic reasoning prompts reveal the potential for large language model interpretability in medicine

**DOI:** 10.1038/s41746-024-01010-1

**Published:** 2024-01-24

**Authors:** Thomas Savage, Ashwin Nayak, Robert Gallo, Ekanath Rangan, Jonathan H. Chen

**Affiliations:** 1 Department of Medicine, Stanford University, Stanford, CA USA; 2 Division of Hospital Medicine, Stanford University, Stanford, CA USA; 3 Palo Alto Veterans Affairs Medical Center, Palo Alto, CA USA; 4 Department of Health Policy, Stanford University, Stanford, CA USA; 5 Stanford Center for Biomedical Informatics Research, Stanford University, Stanford, CA USA; 6 Clinical Excellence Research Center, Stanford University, Stanford, CA USA

**Keywords:** Preclinical research, Diagnosis

## Abstract

One of the major barriers to using large language models (LLMs) in medicine is the perception they use uninterpretable methods to make clinical decisions that are inherently different from the cognitive processes of clinicians. In this manuscript we develop diagnostic reasoning prompts to study whether LLMs can imitate clinical reasoning while accurately forming a diagnosis. We find that GPT-4 can be prompted to mimic the common clinical reasoning processes of clinicians without sacrificing diagnostic accuracy. This is significant because an LLM that can imitate clinical reasoning to provide an interpretable rationale offers physicians a means to evaluate whether an LLMs response is likely correct and can be trusted for patient care. Prompting methods that use diagnostic reasoning have the potential to mitigate the “black box” limitations of LLMs, bringing them one step closer to safe and effective use in medicine.

## Introduction

Large language models (LLMs) are artificial intelligence systems trained on large amounts of text data that learn complex language patterns and syntactical relationships to both interpret passages and generate text output^1,2^ LLMs have received widespread attention for their human-like performance on a wide variety of text-generating tasks. Within medicine, initial efforts have demonstrated that LLMs can write clinical notes^3^, pass standardized medical exams^4^, and draft responses to patient questions^5,6^. In order to integrate LLMs more directly into clinical care, it is imperative to better understand their clinical reasoning capabilities.

Clinical reasoning is a set of problem-solving processes specifically designed for diagnosis and management of a patient’s medical condition. Commonly used diagnostic techniques include differential diagnosis formation, intuitive reasoning, analytical reasoning, and Bayesian inference. Early assessments of the clinical reasoning abilities of LLMs have been limited, studying model responses to multiple-choice questions^7–11^. More recent work has focused on free-response clinical questions and suggests that newer LLMs, such as GPT-4, show promise in diagnosis of challenging clinical cases^12,13^.

Prompt engineering is emerging as a discipline in response to the phenomena that LLMs can perform substantially differently depending on how questions and prompts are posed to them^14,15^. Advanced prompting techniques have demonstrated improved performance on a range of tasks^16^, while also providing insight into how LLMs came to a conclusion (as demonstrated by Wei et al. and Lightman et al. in arithmetic reasoning, common sense reasoning, and symbolic reasoning)^17,18^. A notable example is Chain-of-thought (CoT) prompting, which involves instructing the LLM to divide its task into smaller reasoning steps and then complete the task step-by-step^17^. Given that clinical reasoning tasks regularly use step-by-step processes, CoT prompts modified to reflect the cognitive processes taught to and utilized by clinicians might elicit better understanding of LLM performance on clinical reasoning tasks.

In this paper we evaluate the performance of GPT-3.5 and GPT-4^19^ on open-ended clinical questions assessing diagnostic reasoning. Specifically, we evaluate LLM performance on a modified MedQA USMLE (United States Medical Licensing Exam) dataset^20^, and further evaluate GPT-4 performance on the diagnostically difficult NEJM (New England Journal of Medicine) case series^21^. We compare traditional CoT prompting with several “diagnostic reasoning” prompts that are modeled after the cognitive processes of differential diagnosis formation, intuitive reasoning, analytical reasoning, and Bayesian inference. This study assesses whether LLMs can imitate clinical reasoning abilities using specialized instructional prompts that combine clinical expertise and advanced prompting methods. We hypothesize GPT models will have superior performance with diagnostic reasoning prompts in comparison to traditional CoT prompting.

A modified version of the MedQA USMLE question dataset was used for this study. Questions were converted to free response by removing the multiple-choice options after the question stem. Only Step 2 and Step 3 USMLE questions were included, as Step 1 questions focus heavily on memorization of facts rather than clinical reasoning skills^10^. Only questions evaluating the task of diagnosing a patient were included to simplify prompt engineering. A training set of 95 questions was used for iterative prompt development and a test set of 518 questions was reserved for evaluation. The full test set can be found in Supplementary Data 1.

GPT-4 performance was also evaluated on the New England Journal of Medicine (NEJM) Case Records series. The NEJM Case Records series is designed as an educational resource for physicians, with each case providing a clinical case description followed by expert analysis of the case with a clinical diagnosis. We included the 310 most recently published cases in this study. Ten cases were excluded because they either did not provide a definitive final diagnosis or exceeded the maximum context length of the GPT-4 API. A full list of all cases included (by title and DOI number) can be found in Supplementary Data 2. For this evaluation, we compared traditional CoT prompting to the highest performing clinical reasoning CoT prompt (differential diagnosis reasoning) on the modified MedQA dataset.

One traditional CoT prompt and four clinical reasoning prompts were developed (differential diagnosis, analytical, Bayesian and intuitive reasoning). Each prompt included two example questions (Table 1) with rationales employing the target reasoning strategy. This is a technique known as few-shot learning^14^. The full prompts used for the MedQA dataset are provided in Table 2; the full prompts used for the NEJM challenge set are provided in Supplementary Note 1.

**Table 1:** Example MedQA questions.

Example Question 1
Shortly after undergoing a bipolar prosthesis for a displaced femoral neck fracture of the left hip acquired after a fall the day before, an 80-year-old woman suddenly develops dyspnea. The surgery under general anesthesia with sevoflurane was uneventful, lasting 98 min, during which the patient maintained oxygen saturation readings of 100% on 8 l of oxygen. She has a history of hypertension, osteoporosis, and osteoarthritis of her right knee. Her medications include ramipril, naproxen, ranitidine, and a multivitamin. She appears cyanotic, drowsy, and is oriented only to person. Her temperature is 38.6 °C (101.5 °F), pulse is 135/min, respirations are 36/min, and blood pressure is 155/95 mm Hg. Pulse oximetry on room air shows an oxygen saturation of 81%. There are several scattered petechiae on the anterior chest wall. Laboratory studies show a hemoglobin concentration of 10.5 g/dl, a leukocyte count of 9000/mm^3^, a platelet count of 145,000/mm^3^, and a creatine kinase of 190 U/l. An ECG shows sinus tachycardia. What is the most likely diagnosis?
Example Question 2
A 55-year-old man comes to the emergency department because of a dry cough and severe chest pain beginning that morning. Two months ago, he was diagnosed with inferior wall myocardial infarction and was treated with stent implantation of the right coronary artery. He has a history of hypertension and hypercholesterolemia. His medications include aspirin, clopidogrel, atorvastatin, and enalapril. His temperature is 38.5Â°C (101.3 °F), pulse is 92/min, respirations are 22/min, and blood pressure is 130/80 mm Hg. Cardiac examination shows a high-pitched scratching sound best heard while sitting upright and during expiration. The remainder of the examination shows no abnormalities. An ECG shows diffuse ST elevations. Serum studies show a troponin I of 0.005 ng/ml (*N* < 0.01). What is the most likely cause of this patient’s symptoms?

Example questions used in all MEDQA prompts provided in Table 2.

**Table 2:** CoT and diagnostic reasoning prompts.

Traditional CoT reasoning prompt	
Traditional chain-of-thought (CoT)	Prompt: *Provide a step-by-step deduction that identifies the correct response* {Example Question 1} Example Rationale 1: The patient had a surgical repair of a displaced femoral neck fracture. The patient has petechiae. The patient has a new oxygen requirement, meaning they are having difficulty with their breathing. This patient most likely has a fat embolism. {Example Question 2} Example Rationale 2: This patient is having chest pain. They recently had a heart attack and has new chest pain, suggesting he may have a problem with his heart. The EKG has diffuse ST elevations and he has a scratching murmur. This patient likely has Dressler Syndrome.
Clinical reasoning prompts	
Differential diagnosis CoT	Prompt: *Use step by step deduction to create a differential diagnosis and then use step by step deduction to determine the correct response*. {Example Question 1} Example Rationale 1: This patient has shortness of breath after a long bone surgery. The differential for this patient is pulmonary embolism, fat embolism, myocardial infarction, blood loss, anaphylaxis, or a drug reaction. The patient has petechiae which makes fat embolism more likely. This patient most likely has a fat embolism. {Example Question 2} Example Rationale 2: This patient has chest pain with diffuse ST elevations after a recent myocardial infarction. The differential for this patient includes: myocardial infarction, pulmonary embolism, pericarditis, Dressler syndrome, aortic dissection, and costochondritis. This patient likely has a high-pitched scratching sound on auscultation associated with pericarditis and Dressler Syndrome. This patient has diffuse ST elevations associated with Dressler Syndrome. This patient most likely has Dressler Syndrome.
Intuitive reasoning CoT	Prompt: *Use symptom, signs, and laboratory disease associations to step by step deduce the correct response*. {Example Question 1} Example Rationale 1: This patient has findings of petechiae, altered mental status, shortness of breath, and recent surgery suggesting a diagnosis of fat emboli. The patient most likely has a fat embolism. {Example Question 2} Example Rationale 2: This patient had a recent myocardial infarction with new development of diffuse ST elevations, chest pain, and a high pitched scratching murmur which are found in Dressler’s syndrome. This patient likely has Dressler’s Syndrome.
Analytic reasoning CoT	Prompt: *Use analytic reasoning to deduce the physiologic or biochemical pathophysiology of the patient and step by step identify the correct response*. {Example Question 1} Example Rationale 1:The patient recently had large bone surgery making fat emboli a potential cause because the bone marrow was manipulated. Petechiae can form in response to capillary inflammation caused by fat emboli. Fat micro globules cause CNS microcirculation occlusion causing confusion and altered mental status. Fat obstruction in the pulmonary arteries can cause tachycardia and shortness of breath as seen in this patient. This patient most likely has a fat embolism. {Example Question 2} Example Rationale 2: This patient had a recent myocardial infarction which can cause myocardial inflammation that causes pericarditis and Dressler Syndrome. The diffuse ST elevations and high pitched scratching murmur can be signs of pericardial inflammation as the inflamed pericardium rubs against the pleura as seen with Dressler Syndrome. This patient likely has Dressler Syndrome.
Bayesian reasoning CoT	Prompt: *Use step-by-step Bayesian Inference to create a prior probability that is updated with new information in the history to produce a posterior probability and determine the final diagnosis*. {Example Question 1} Example Rationale 1: The prior probability of fat embolism is 0.05% however the patient has petechiae on exam which is seen with fat emboli, which increases the posterior probability of fat embolism to 5%. Altered mental status increases the probability further to 10%. Recent orthopedic surgery increases the probability of fat emboli syndrome to 60%. This patient most likely has a fat embolism. {Example Question 2} Example Rationale 2: The prior probability of Dressler Syndrome is 0.01%. The patient has diffuse ST elevations, increasing the probability of Dressler Syndrome to 5%. The patient has a scratching murmur which increases the probability to 10%. In the setting of a recent MI the posterior probability of myocardial infarction is 55%. This patient likely has Dressler Syndrome.

The traditional and diagnostic CoT prompts evaluated in this study. The provided rationales are in response to the example questions provided in Table 1. Note that the full text of the example questions has been omitted for readability.

**Fig. 1:**
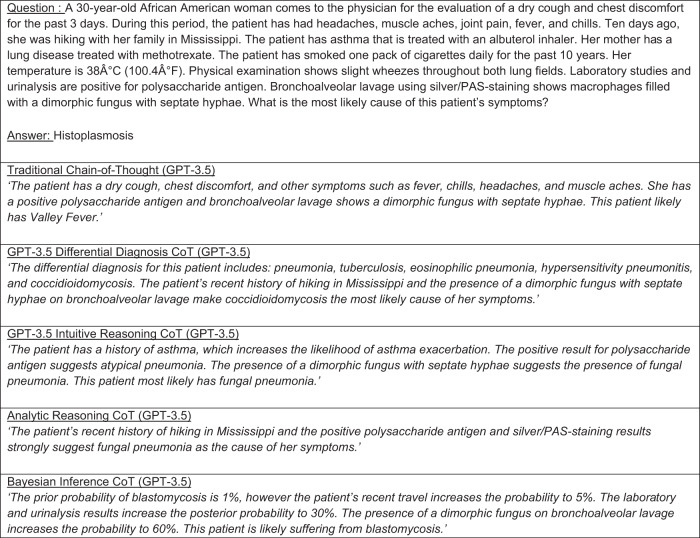
GPT 3.5 CoT and diagnostic reasoning rationale examples. Example GPT-3.5 rationales responding to a traditional CoT prompt as well as diagnostic reasoning prompts. LLM response and rationale results for the entire test set can be found in Supplementary Information 1.

**Fig. 2:**
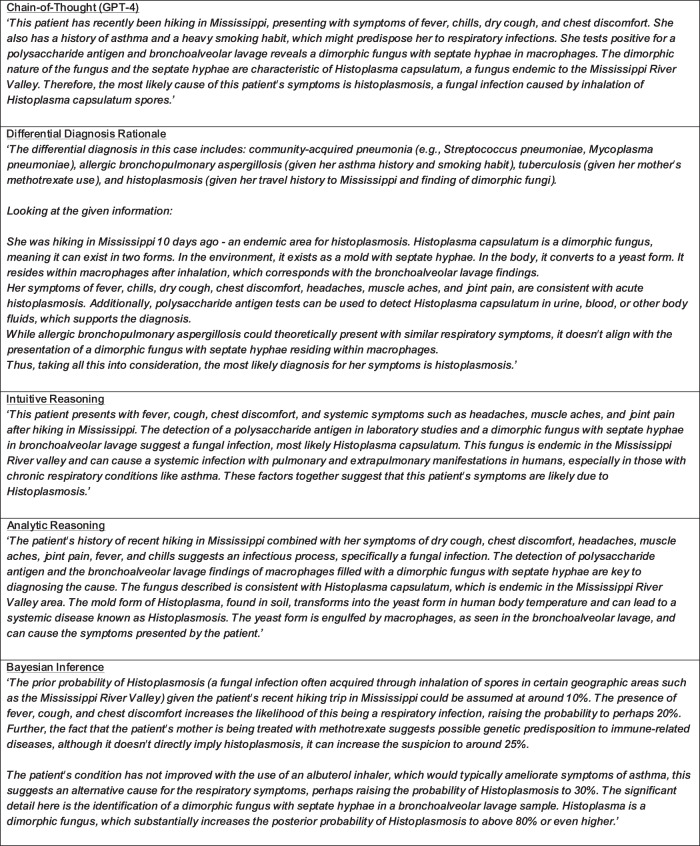
GPT 4 CoT and diagnostic reasoning rationale examples. Example GPT-4 rationales responding to the question posed in Fig. 1. LLM response and rationale results for the entire test set can be found in Supplementary Information 1.

Example LLM responses for each prompting strategy can be found in Fig. 1 for GPT-3.5 and Fig. 2 for GPT-4. Full results can be found in Supplementary Data 1 and 2.

## Results

GPT-3.5 correctly answered 46% of questions using traditional CoT prompting, compared to 31% with zero-shot non-CoT prompting. Among the clinical reasoning prompts, GPT-3.5 achieved the highest performance with intuitive reasoning (48% vs. 46%, difference of +1.7%, CI −2.5% to +5.9%, *p* = 0.4). Compared to traditional CoT, GPT-3.5’s performance was significantly worse with analytic reasoning (40%, difference of −6%, CI −11% to −1.5%, *p* = 0.001) and differential diagnosis formation (38%, difference of −8.9%, CI −14% to −3.4%, *p* = <0.001), while Bayesian inference performance nearly missed our threshold for statistical significance (42%, difference of −4.4%, CI −9.1% to +0.2%, *p* = 0.02). Results can be referenced in Table 3. Inter-rater agreement for the MedQA GPT-3.5 evaluation was 97% with a Cohen’s Kappa of 0.93.

**Table 3:** GPT 3.5 MEDQA performance with diagnostic reasoning prompts compared to traditional CoT.

Prompt	Correct responses (%)	Difference in percentage (confidence interval)	*p* value^a^
Chain of thought	46%	–	–
Intuitive reasoning	48%	1.7% (−2.5%, 5.9%)	0.4
Analytic reasoning	40%	−6.0% (−11%, −1.5%)	0.001
Differential diagnosis	38%	−8.9% (−14%, −3.4%)	<0.001
Bayesian inference	42%	−4.4% (−9.1%, 0.2%)	0.02

GPT-3.5 performance on a free-response MEDQA question set with both traditional chain-of-thought model prompting strategies as well as clinical reasoning prompts of intuitive reasoning, analytic reasoning, differential diagnosis and Bayesian inference.

^a^Percentage difference and *p* value statistics compared to traditional chain-of-thought.

The GPT-4 API generated an error for 20 questions of the test set, reducing the test set size to 498. Overall, GPT-4 demonstrated improved accuracy over GPT-3.5. GPT-4 achieved an accuracy of 76% with traditional CoT, 77% with intuitive reasoning (+0.8%, CI −3.6% to +5.2%, *p* = 0.73), 78% with differential diagnosis (+2.2%, CI −2.3% to +6.7%, *p* = 0.24), 78% with analytic reasoning (+1.6%, CI −2.4% to +5.6%, *p* = 0.35), and 72% with Bayesian Inference (−3.4%, CI −9.1% to +1.2%, *p* = 0.07). Results can be found in Table 4. Inter-rater agreement for the GPT-4 MedQA evaluation was 99% with a Cohen’s Kappa of 0.98.

**Table 4:** GPT 4 MEDQA performance with diagnostic reasoning prompts compared to traditional CoT.

Prompt	Correct responses (%)	Difference in percentage (confidence interval)	*p* value^a^
Chain of thought	76%	–	–
Intuitive reasoning	77%	0.8% (−3.6%, 5.2%)	0.73
Analytic reasoning	78%	1.6% (−2.4%, 5.6%)	0.35
Differential diagnosis	78%	2.2% (−2.3%, 6.7%)	0.24
Bayesian inference	72%	−3.4% (−9.1%, 1.2%)	0.07

GPT-4 performance on a free-response MEDQA question set with both traditional chain-of-thought model prompting strategies as well as clinical reasoning prompts of intuitive reasoning, analytic reasoning, differential diagnosis and Bayesian inference.

^a^Percentage difference and *p* value statistics compared to traditional chain-of-thought.

On the NEJM challenge case set GPT-4 achieved an accuracy of 38% with traditional CoT compared to 34% with differential diagnosis CoT (difference of −4.2%, 95% CI −11.4% to +2.1%, *p* = 0.09, Table 5). Inter-rater agreement for the GPT-4 NEJM evaluation was 97% with a Cohen’s Kappa of 0.93. GPT-4 response and rationale results for the entire NEJM test set are included in Supplementary Data 2.

**Table 5:** GPT 4 challenge set performance with differential diagnosis reasoning prompts compared to traditional CoT.

Prompt	Correct responses (%)	Difference in percentage (confidence interval)	*p* value
Chain of thought	38%	–	–
Differential diagnosis	34%	−4.2% (−11.4%, +2.1%)	0.09

GPT-4 performance on the NEJM challenge question set with both traditional chain-of-thought and differential diagnosis reasoning prompting.

## Discussion

In this study we found that GPT-3.5 performance was similar with traditional and intuitive reasoning CoT prompts, but significantly worse with differential diagnosis and analytical CoT prompts. Bayesian inference CoT also demonstrated worse performance than traditional CoT, but the decrease in performance did not meet our significance threshold. These findings suggest GPT-3.5 is not able to imitate advanced clinical reasoning processes to arrive at an accurate diagnosis. In contrast, GPT-4 demonstrated similar performance between traditional and diagnostic reasoning CoT prompts. While these findings highlight the significant advancement in reasoning abilities between GPT-3.5 and GPT-4, diagnostic reasoning does not increase GPT-4 accuracy like it would for a human provider. We propose three possible explanations for this finding. First, GPT-4’s reasoning mechanisms could be inherently different than human providers and therefore does not derive benefit from diagnostic reasoning strategies. Second, GPT-4 could be explaining its diagnostic evaluation post-hoc in the desired diagnostic reasoning format instead of strictly using the prompted diagnostic reasoning strategy. Third, GPT-4 could have reached a maximal accuracy with the vignette information provided and we are thus unable to detect an accuracy difference between prompting strategies. Regardless of the underlying reason, we observe GPT-4 has developed the ability to successfully imitate clinical reasoning thought processes but cannot apply clinical reasoning like a human.

**Fig. 3:**
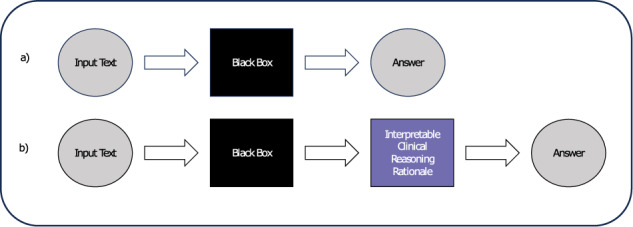
Proposed LLM workflow. **a** Current LLM workflow. **b** Proposed LLM workflow.

The finding that GPT-4 can successfully imitate the same cognitive processes as physicians to arrive accurately at an answer is still significant because of the potential for interpretability. We define interpretability as the property that allows a human operator to explore qualitative relationships between inputs and outputs^22^. A model that generates a clinical reasoning rationale when suggesting a diagnosis offers the clinician an interpretable means to assess whether the answer is true or false based on the rationale’s factual and logical accuracy. A workflow that aligns model outputs in this way (Fig. 3) could mitigate the “black box” limitations of LLMs, as long as physicians recognize that language models will always be at risk of unpredictable reasoning hallucinations, and that rationale logical and factual accuracy still does not absolutely guarantee answer correctness.

To demonstrate how clinical reasoning prompts provide interpretability, we include descriptive MedQA examples (Supplementary Data 4). Incorrect model responses are often accompanied by rationales that provide factual inaccuracy, while logical rationales are more often associated with correct responses. We further quantify this relationship by evaluating 100 GPT-4 diagnostic reasoning rationales, where we found incorrect answers were much more likely to have logic errors in their rationale compared to correct answers. In total, 65% of incorrect answers had false logic statements in their rationale, with an average of 0.82 inaccuracies per rationale. In contrast, only 18% of correct answers had false logic statements in their rationale, with an average of 0.11 per question (Supplementary Data 5). Our results suggest clinical reasoning rationales provide valuable insight (but not an absolute guarantee) into whether an LLM response can be trusted and represent a step toward LLM interpretability.

The strengths of our investigation are a prompt design that leverages chain-of-thought prompting for insight into LLM clinical reasoning capabilities as well as the use of free response clinical case questions where previous studies have been limited to multiple-choice or simple open-ended fact retrieval that do not challenge LLM clinical reasoning abilities. We designed our evaluation with free response questions both from the USMLE as well as NEJM case report series to facilitate rigorous comparison between prompting strategies.

A limitation of our study is that while our prompt engineering process surveyed a wide range of prompt styles we could not test all possible diagnostic reasoning CoT prompts. Furthermore our investigation was limited to only GPT-3.5 and GPT-4, US-centric question sets, and the English language, therefore we cannot generalize our findings to other available models, especially ones fine-tuned on texts demonstrating clinical reasoning, nor to non-English languages and non-US-centric question sets. We hope that future studies can iterate on our diagnostic reasoning prompts and use our open dataset as a benchmark for additional evaluation.

## Methods

### LLM prompt development

We used an iterative process known as prompt engineering to develop our diagnostic reasoning prompts. During this process, we experimented with several different types of prompts (Supplementary Note 2). In each round of prompt engineering, we evaluated GPT-3.5 accuracy on the MEDQA training set (Supplementary Data 3). We found prompts that encouraged step-by-step reasoning without specifying what the steps should be, yielded better performance. We also found that prompts that focused on a single diagnostic reasoning strategy provided better results than prompts that combined multiple strategies.

### LLM response evaluation

Language model responses were evaluated by physician authors AN, ER, RG and TS, three internal medicine attending physicians and one internal medicine resident. Each question was evaluated by two blinded physicians. If there was disagreement in the grade assigned, a third evaluator determined the final grade. Any response that was felt to be equally correct and specific, as compared to the provided answer, was marked as correct. Physicians used UpToDate^23^, MKSAPP^24^, and StatPearls^25^ to verify accuracy of answers when needed.

### LLM programming and computing resources

For this evaluation we used the OpenAI Davinci-003 model via an OpenAI API to provide GPT-3.5 responses and GPT-4 model via an OpenAI API to provide GPT-4 responses. Prompting of the GPT-3.5 model was performed with the Demonstrate-Search-Predict (DSP) Python module^26,27^. Self-consistency was applied to all GPT-3.5 Chain-of-Thought prompts^28^. GPT-4 responses did not use DSP or self-consistency because those features were not available for GPT-4 at the time of submission. Computing was performed in a Google CoLab Jupyter Notebook. Full code can be found in Supplementary Note 3.

### Statistical evaluation

Statistical significance and confidence intervals were calculated against traditional CoT using McNemar’s test for paired proportions, two-tailed. Statistical significance was set at an alpha of 0.0125 to reflect multiple hypotheses (four prompts per each model) by the Bonferroni Correction. Inter-rater agreement was assessed using Cohen’s Kappa Statistic. Statistical analysis was performed in R with the epibasix library.

### Clinical reasoning rationale logic evaluation

The first 100 GPT-4 differential diagnosis rationales were evaluated for appropriate logic and medical accuracy. The rationales were evaluated by physician authors RG and TS, who are both internal medicine attending physicians.

The reviewers attempted to identify instances of inaccuracy or false logic in each diagnostic reasoning rationale, blinded to the index question, gold standard answer, or grade of the LLM response. Reviewers were blinded to the index question to simulate a clinical situation where a physician is evaluating an LLM case interpretation without examining the patient themselves. Arguments with false logic or inaccuracies were tallied and a comparison was made between rationales supporting correct versus incorrect answers. Complete data can be found in Supplementary Data 5.

### Supplementary information

Supplementary Information

Supplementary Data 1

Supplementary Data 2

Supplementary Data 3

Supplementary Note 3 Part 1

Supplementary Note 3 Part 2

Supplementary Table 1

Supplementary Table 2

Supplementary Data 5

## Data Availability

All data used in this manuscript are provided in our Supplementary Information and open access figshare (10.6084/m9.figshare.24886593). This includes all prompts, LLM responses and reviewer grades.

## Code availability

All code used in this manuscript is provided in our Supplementary Information and open access figshare (10.6084/m9.figshare.24886593).
