# Supplementary material for: Diagnostic reasoning prompts reveal the potential for large language model interpretability in medicine
Source: NPJ Digit Med. 2024 Jan 24;7:20. doi: 10.1038/s41746-024-01010-1 (PMC10808088; doi:10.1038/s41746-024-01010-1)
Supplement: Supplementary file 1 — Supplementary Information [file 41746_2024_1010_MOESM1_ESM.docx]

### Supplementary Information

**Supplementary Data 1**

See file “MedQA_complete_graded_data.csv” for our complete test question set and prompt responses with grades for the MedQA evaluation of GPT-3.5 and GPT-4.

**Supplementary Data 2**

See file “NEJM_complete_graded_data.csv” for our complete test question set and prompt responses with grades for the NEJM clinical challenge evaluation of GPT-4.

**Supplementary Note 1**

Below are the prompts used for the NEJM challenge cases. Each prompt includes a question-answer-rationale example based on DOI 10.1056/NEJMcpc1413303. The text for DOI 10.1056/NEJMcpc1413303 is not included in this Supplementary Information to respect copyright. These prompts were submitted to the GPT-4 API via the code provided in Supplementary Note 3.

**Traditional Chain of Thought**

Read the initial presentation of a medical case below and determine the final diagnosis. Assume that all of the relevant details from figures and tables have been explained in the text. When providing your rationale, USE STEP-BY-STEP DEDUCTION TO IDENTIFY THE CORRECT RESPONSE. After you provide your rationale, provide a single, specific diagnosis for the case in less than 10 words.

Example Case:

DOI 10.1056/NEJMcpc1413303 Text

Rationale(REMEMBER TO USE STEP BY STEP DEDUCTION):

This patient has oral ulcers, which can be associated with autoimmune and infectious diseases. The patient has a negative infectious work up and did not respond to antibiotics, which supports an autoimmune process. The patient has genital ulcers, which are associated with the autoimmune process of Behcets disease. Nodules of the legs further support an autoimmune process such as Behcets disease. Symmetric arthralgias are seen in a majority of patients with Behcets disease. Fever can be seen in systemic autoimmune processes such as Behcets disease. The rash is described as pustular, which can be seen in pathergy phenomenon, a highly specific sign for Behcets disease.

Diagnosis:

Behcets disease is the most likely diagnosis.

===

Case:

**Case Text Provided **

Rationale (REMEMBER TO USE STEP BY STEP DEDUCTION):

Diagnosis:

**Differential Diagnosis Formation**

Read the initial presentation of a medical case below and determine the final diagnosis. Assume that all of the relevant details from figures and tables have been explained in the text. When providing your rationale, USE STEP BY STEP DEDUCTION TO CREATE A DIFFERENTIAL DIAGNOSIS AND THEN USE STEP BY STEP DEDUCTION TO DETERMINE THE CORRECT RESPONSE. After you provide your rationale, provide a single, specific diagnosis for the case in less than 10 words.

Example Case:

DOI 10.1056/NEJMcpc1413303 Text

Rationale (REMEMBER TO CREATE A DIFFERENTIAL DIAGNOSIS):

This is a young man who presents with oral ulcers, odynophagia, rash, and joint pains. He also has genital ulcers and fever. These findings support either an autoimmune or infectious process. The patient has a family history of autoimmune disease (father has Ulcerative Colitis) and infectious risk factors of unprotected sex and recent travel to Hawaii. The differential diagnosis for this patient includes sexually transmitted infections, Crohn’s disease, Behcet’s disease and Lupus. The patient’s urinalysis is normal, making chlamydia or gonorrhea less likely despite a history of unprotected sex. Furthermore, the rash described is pustular with papules and plaques of the trunk, thighs, and buttocks, as well as nodular of the shins without fluctuance. This is not the characteristic rash of Syphilis or Herpes simplex, removing sexually transmitted infections from our differential. The rash description is also not characteristic of lupus and the patient does not have evidence of kidney disease. The patient is also a young male. This makes Lupus less likely. The patient’s does not have diarrhea or gastrointestinal symptoms that would be consistent with Crohn’s disease, despite a family history of Ulcerative Colitis. This makes Crohn’s disease less likely. The constellation of oral ulcers, genital ulcers, and a pustular rash in a young male is concerning for Behcet’s disease. Furthermore, the patient’s other non-specific symptoms of rash, fever, and joint pains are supportive of an autoimmune process.

Diagnosis:

Behcet’s disease is the most likely diagnosis.

===

Case:

**Case Text Provided **

Rationale (REMEMBER TO CREATE A DIFFERENTIAL DIAGNOSIS):

Diagnosis:

**Supplementary Note 2**

In the process of engineering our clinical reasoning prompts, we experimented with many different prompting strategies. All prompts tested are included below. The first prompt listed of every section is the final prompt that was selected. The number in parentheses is the number of questions the prompt answered correctly from the development set. Full results of our development set evaluation are found in Supplementary Data 3.

Intuitive Reasoning

1. Use symptom, signs, and laboratory disease associations to step by step deduce the correct response. (39)
2. Use disease association to deduce the correct response. (38)
3. First list the top 6 diagnoses that answer the question. Then reference the question to find patient information (past medical history, symptoms, physical exam findings, lab tests, imaging) that are associated with any of the diagnoses in the differential. Answer the question based on the diagnoses that are most likely. (39)
4. Create a differential diagnosis and then use disease association to deduce the correct response. (39)
5. First list the top 6 diagnoses that answer the question. Then reference the question to find patient information or test results that are associated with any of the diagnoses in the differential. Answer with the diagnosis that is most likely. (39)
6. Follow the following steps: 1) list a broad differential of 6 diagnoses that answer the question. 2) reference the question to find patient information or test results that make certain diagnoses on the differential more likely. 3) Narrow the differential to 3 diagnoses based. 4) Again reference the question to find information that makes one diagnosis more likely. 6) Answer with the most likely diagnosis. (39)

Analytic Reasoning

1. Use analytic reasoning to deduce the physiologic or biochemical pathophysiology of the patient and step by step identify the correct response. (40)
2. Use analytic reasoning to deduce the physiologic or biochemical pathophysiology of the patient and identify the correct response. (30)
3. Systematically reference each piece of patient information or test result in the prompt, explain if and how each piece of information supports one diagnosis. Answer with the most likely diagnosis. (30)
4. First list the top 6 diagnoses that answer the question. Then systematically reference each piece of patient information or test result in the prompt, explain if and how each piece of information supports one of the diagnoses on the differential. Answer with the most likely diagnosis. (33)
5. First list the top 6 diagnoses that answer the question. Then systematically reference each piece of information in the prompt (past medical history, symptoms, physical exam findings, lab tests, imaging), explain if and how each piece of information supports one of the diagnoses on the differential. Select the diagnosis most likely based on which diagnosis is most supported. (32)
6. Create a differential diagnosis, then use analytic reasoning to deduce the physiologic or biochemical pathophysiology of the patient and identify the correct response. (35)

Bayesian Inference

1. Use step-by-step Bayesian Inference to create a prior probability that is updated with new information in the history to produce a posterior probability and determine the final diagnosis. (48)
2. Calculate a Bayes prior probability for the most likely diagnosis. Then reference the question to find all important patient information (past medical history, symptoms, physical exam findings, lab tests, imaging) that helps determine the diagnosis. For each piece of information, estimate the likelihood of the diagnosis being considered and calculate a posterior probability. Select the most likely diagnosis. (35)
3. First list the top 6 diagnoses that answer the question. Calculate a Bayes prior probability for each diagnosis. Then reference the question to find all important patient information or test results that helps determine the diagnosis. For each piece of information, estimate a likelihood of this information with each diagnosis being considered. Calculate a posterior probability for each diagnosis. Select the answer with the highest posterior probability. (39)
4. Create a differential diagnosis, then use a step-by-step Bayesian inference to deduce the correct response. (41)

Differential Diagnosis

1. Use step by step deduction to create a differential diagnosis and then use step by step deduction to determine the correct response. (33)
2. First list the top 6 diagnoses that answer the question, select the diagnosis that is most likely. (30)
3. Create a differential diagnosis and use step by step deduction to determine the correct response. (33)
4. Follow the following steps: 1) list a broad differential of 6 diagnoses that answer the question. 2) reference the question to find patient information or test results that make certain diagnoses on the differential more likely. 3) Narrow the differential to 3 diagnoses based. 4) Again reference the question to find information that makes one diagnosis more likely. 6) Answer with the most likely diagnosis. (33)

**Supplementary Data 3**

See file “Dev_Results.csv” for complete results of the Supplementary Note 2 prompts on our training set.

**Supplementary Note 3**

See file “GPT3.5_API.ipynb” for our complete code for submission of prompts to the GPT-Davinici-003 API.

See file “GPT4_API.ipynb” for our complete code for submission of prompts to the GPT-4 API.

**Supplementary Data 4**

Supplementary Table 1 and 2 exhibits GPT-4 MedQA examples where the model incorrectly answered the question and false logic can be identified in the rationale. Each example demonstrates how clinical reasoning rationales can provide model interpretability.

**Supplementary Data 5**

See file “GPT4_Plausibility_test.xlsx” for complete results of our clinical reasoning rationale logic evaluation.

=========================

Supplementary files link: https://drive.google.com/drive/folders/1mDQUZ4RhyROSEycVFN_c4uyP36oyMRSe?usp=sharing
