# Supplementary material for: Diagnostic reasoning prompts reveal the potential for large language model interpretability in medicine
Source: NPJ Digit Med. 2024 Jan 24;7:20. doi: 10.1038/s41746-024-01010-1 (PMC10808088; doi:10.1038/s41746-024-01010-1)
Supplement: Supplementary file 7 — Supplementary Table 1 [file 41746_2024_1010_MOESM7_ESM.docx]

| **Question 1**  A previously healthy 15-year-old girl is brought to the emergency department 24 hours after the onset of a severe headache. She returned from a 1-week camping trip 3 days ago; she went spelunking and swimming in a freshwater lake during the trip. She is agitated, uncooperative, and oriented only to person. Her temperature is 38.9 C (102 F), pulse is 112/min, respirations are 20/min, and blood pressure is 100/68 mm Hg. There are several crusted insect bites on her extremities. Neurologic examination shows diffuse hyperreflexia and an extensor plantar response bilaterally. Her neck is supple without lymphadenopathy. An MRI of the brain shows asymmetrical, bitemporal hyperintensities. A lumbar puncture is performed. Cerebrospinal fluid analysis shows:  Opening pressure 150 mm H2O  Glucose 58 mg/dLx  Protein 108 mg/dL  Leukocyte count 150/mm3  Segmented neutrophils 15%  Lymphocytes 85%  Erythrocyte count 25/mm3  What is the most likely causal pathogen?  **Rationale** (Intuitive Reasoning):  The patient's recent history of a camping trip with spelunking and swimming in a freshwater lake, in conjunction with her symptoms, which include severe headache, agitation, hyperreflexia, and an extensor plantar response bilaterally, suggest a central nervous system infection. The MRI findings of asymmetrical, bitemporal hyperintensities suggest encephalitis rather than meningitis, given the involvement of brain parenchyma. The cerebrospinal fluid findings of mildly elevated protein and lymphocytic pleocytosis also align with a viral cause of encephalitis. Among viruses, the Naegleria fowleri is notable for its association with freshwater exposure, particularly in warm water, and for causing primary amebic meningoencephalitis, a severe and often fatal brain infection. The rapid onset and severity of symptoms in this previously healthy girl are also typical of infections with this free-living ameba.  **Model Answer**: Naegleria Fowleri **Correct Answer:** HSV  **Incorrect Logic**: Naegleria Fowleri is not a virus but an amoeba. Naegleria Fowleri CSF studies typically show a high WBC with predominantly PMNs rather than lymphocytes. |
| --- |
| **Question 2**  A 32-year-old G1P0 woman presents to her obstetrician for a prenatal visit. She is 30 weeks pregnant. She reports some fatigue and complains of urinary urgency. Prior to this pregnancy, she had no significant medical history. She takes a prenatal vitamin and folate supplements daily. Her mother has diabetes, and her brother has coronary artery disease. On physical examination, the fundal height is 25 centimeters. A fetal ultrasound shows a proportional reduction in head circumference, trunk size, and limb length. What is the most likely cause of the patient’s presentation?  **Rationale** (Differential Diagnosis):  Rationale: In this case, the differential diagnoses for reduced fetal size include intrauterine growth restriction (IUGR), congenital infections, and chromosomal abnormalities. IUGR can be a result of placental insufficiency, maternal malnutrition, maternal illnesses (such as hypertension or diabetes), or certain medications. Congenital infections that can lead to reduced fetal size include TORCH infections (Toxoplasmosis, Other agents, Rubella, Cytomegalovirus, and Herpes simplex). Certain chromosomal abnormalities, such as Down syndrome and Turner syndrome, can also result in smaller babies.  This patient presents with a proportional reduction in fetal size (head circumference, trunk, and limbs), which is more consistent with constitutional growth delay (the baby is small but proportional), rather than a specific cause such as a congenital infection or chromosomal abnormalities which often present with certain specific features.  Furthermore, the patient doesn't mention any symptoms suggestive of an infection nor any risk factors for chromosomal abnormalities (e.g., advanced maternal age). There's no mention of significant maternal disease, drug use, or malnutrition. However, it's mentioned that the patient's mother has diabetes and her brother has coronary artery disease. These familial factors could hint towards a genetic predisposition for metabolic issues which could impact fetal growth.  Therefore, the most likely cause of this patient's presentation is constitutional growth delay due to genetic factors.  **Model Answer:** Genetic factors. **Correct Answer**: Rubella  **Incorrect Logic**: Gestational diabetes and familial history of coronary artery disease are not associated with genetic factors that cause intrauterine growth restriction. |
| **Question 3**  A 47-year-old man presents to the clinic for an evaluation of intense itching of his right thigh region for the past few days. He states some ‚ bumps just began to form. The patient mentions that he was recently at a business conference in Miami. He has a past medical history of hypertension, diabetes type 2, and hyperlipidemia. He takes enalapril, metformin, and atorvastatin. He does not smoke or drink. His vitals are within normal limits today. On physical examination, a linear line with 3 red papules is present along the medial aspect of his right thigh. Additionally, there are small rows of bumps on his left leg and right forearm. Excoriations are also apparent in the same region. What is the most likely diagnosis?  **Rationale** (Analytic Reasoning):  The patient presents with intense itching, red bumps, and a linear line with 3 red papules on the medial aspect of his right thigh, as well as small rows of bumps on his left leg and right forearm. His recent travel to Miami, a region known for its humid climate and prevalence of insects, hints at a potential exposure to pests. The pattern of skin lesions suggests that this is not a systemic allergic or inflammatory reaction, but rather a result of localized external factors. The linear arrangement of the papules is characteristic of a path where an arthropod, such as a mite, might have burrowed into the skin. The intense itching and red papules are indicative of an inflammatory reaction to the mite and its products. The clinical presentation is highly suggestive of scabies, which is caused by the mite Sarcoptes scabiei burrowing into the skin and laying eggs, leading to an intensely pruritic rash with characteristic burrows.  **Model Answer:** Scabies **Correct Answer**: Bed Bugs  **Incorrect Logic**: Linear bite marks are characteristic of bed bugs, not scabies, and the location of bites on the thigh, let and forearm are also atypical for scabies and more classic for bed bugs. |
| **Question 4**  A 17-year-old boy is brought to the physician because of progressive right knee pain for the past 3 months. He reports that the pain is worse at night and while doing sports at school. He has not had any trauma to the knee or any previous problems with his joints. His vital signs are within normal limits. Examination of the right knee shows mild swelling and tenderness without warmth or erythema; the range of motion is limited. He walks with an antalgic gait. Laboratory studies show an alkaline phosphatase of 180 U/L and an erythrocyte sedimentation rate of 80 mm/h. An x-ray of the right knee is shown. What is the most likely diagnosis?  **Rationale** (Bayesian Inference):  The prior probability of Osgood-Schlatter disease in an adolescent male presenting with knee pain is around 10%. However, the patient has been experiencing pain for the past 3 months and the pain is worse during sports, this would increase the probability to about 20%. Since there is no history of trauma and the patient is in adolescence, this increases the likelihood of Osgood-Schlatter disease, pushing the probability to 30%. The presence of mild swelling and tenderness without warmth or erythema, and an antalgic gait further support the diagnosis, increasing the probability to 50%. The elevated alkaline phosphatase and erythrocyte sedimentation rate are also consistent with Osgood-Schlatter disease, elevating the probability to 60%. Considering all these features, Osgood-Schlatter disease is the most likely diagnosis.  **Model Answer**: Osgood Schlatter **Correct Answer**: Osteosarcoma  **Incorrect Logic**: Alkaline phosphatase and Erythrocyte Sedimentation Rate (ESR) are not classically elevated with Osgood-Schlatter disease. |
| **Question 5**  A previously healthy 13-year-old girl is brought to the physician by her mother because of a change in behavior. The mother reports that over the past 6 months, her daughter has had frequent mood swings. Sometimes, she is irritable for several days and loses her temper easily. In between these episodes, she behaves normal and spends time with her friends, and participates in gymnastics training twice a week. The mother has also noticed that her daughter needs more time than usual to get ready for school. Sometimes, she puts on excessive make-up. One month ago, her teacher had informed the parents that their daughter had skipped school and was seen at the local mall with one of her classmates instead. The patient reports that she often feels tired, especially when she has to wake up early for school. On the weekends, she sleeps until 1 pm. Menses have occurred at 15- to 45-day intervals since menarche at the age of 12 years; they are not associated with abdominal discomfort or functional impairment. Physical examination shows no abnormalities. What is the most likely explanation for the patient's behavior?  **Rationale** (Intuitive Reasoning):  The patient, a 13-year-old girl, has presented with recent changes in her behavior including frequent mood swings, periods of irritability, needing more time to get ready (possibly a sign of perfectionism or obsessive behavior), skipping school, and excessive sleepiness. These signs may suggest an underlying psychiatric condition. Considering the age of onset, her mood swings, and changes in behavior and sleep pattern, it is suggestive of a mood disorder. Bipolar disorder can present in adolescence and may include periods of depression (indicated here by sleeping in, fatigue, and possible social withdrawal) interspersed with periods of elevated, expansive, or irritable mood. The use of excessive makeup and the act of skipping school may reflect impulsive or risk-taking behavior, which can be seen in the manic or hypomanic phase of bipolar disorder.  **Model Answer:** Bipolar Disorder **Correct Answer**: Normal Adolescent Behavior  **Incorrect Logic**: Applying excessive makeup or skipping school in a young teenage girl is not a sign of manic or hypomanic behavior and is more consistent with normal adolescent behavior. |
| **Question 6**  A newborn infant presents with severe weakness. He was born to a G1P1 mother at 40 weeks gestation with the pregnancy attended by a midwife. The mother's past medical history is unremarkable. She took a prenatal vitamin and folic acid throughout the pregnancy. Since birth, the child has had trouble breastfeeding despite proper counseling. He also has had poor muscle tone and a weak cry. His temperature is 99.5 F (37.5 C), blood pressure is 57/38 mmHg, pulse is 150/min, respirations are 37/min, and oxygen saturation is 96% on room air. Physical exam reveals poor muscle tone. The patient's sucking reflex is weak, and an enlarged tongue is noted. An ultrasound is performed, and is notable for hypertrophy of the myocardium. What is the most likely diagnosis?  **Rationale** (Differential Diagnosis Reasoning):  The differential diagnosis for a newborn presenting with severe weakness, poor feeding, enlarged tongue, poor muscle tone and hypertrophic cardiomyopathy includes: congenital myopathies, metabolic disorders, spinal muscular atrophy, and congenital hypothyroidism. Considering the symptomatology, congenital hypothyroidism and metabolic disorders are less likely as they typically do not cause hypertrophic cardiomyopathy. Spinal muscular atrophy is a possibility; however, hypertrophic cardiomyopathy is not typically seen in this condition. The most likely diagnosis in this case is a congenital myopathy, more specifically, infantile botulism due to the presence of a weak cry, poor sucking reflex, generalized weakness, and hypertrophic cardiomyopathy.  **Model Answer**: Infantile Botulism **Correct Answer**: Acid Maltase deficiency  **Incorrect Logic**: Infantile Botulism is not a form of congenital myopathy and is not associated with hypertrophic cardiomyopathy. |
| **Question 7**  A 67-year-old man is brought to the emergency department when he was found obtunded at the homeless shelter. The patient is currently not responsive and smells of alcohol. The patient has a past medical history of alcohol use, IV drug use, and hepatitis C. His temperature is 99¬∞F (37.2¬∞C), blood pressure is 95/65 mmHg, pulse is 95/min, respirations are 13/min, and oxygen saturation is 95% on room air. The patient is started on IV fluids, and his pulse decreases to 70/min. On physical exam, the patient has an abdominal exam notable for distension and a positive fluid wave. The patient displays mild yellow discoloration of his skin. The patient has notable poor dentition and poor hygiene overall. A systolic murmur is heard along the left sternal border on cardiac exam. Pulmonary exam is notable for mild bibasilar crackles. Laboratory values are ordered, and return as below:  Hemoglobin: 10 g/dL  Hematocrit: 32%  Leukocyte count: 7,500 cells/mm^3 with normal differential  Platelet count: 227,000/mm^3  Serum:  Na+: 125 mEq/L  Cl-: 100 mEq/L  K+: 5.0 mEq/L  HCO3-: 24 mEq/L  BUN: 51 mg/dL  Glucose: 89 mg/dL  Creatinine: 2.2 mg/dL  Ca2+: 10.0 mg/dL  AST: 22 U/L  ALT: 19 U/L  Urine:  Color: Amber  Nitrites: Negative  Sodium: 12 mmol/24 hours  Red blood cells: 0/hpf  Over the next 24 hours, the patient produces very little urine. What best explains this patients renal findings?  **Rationale** (Differential Diagnosis Rationale):  The differential diagnosis for this patient includes dehydration, acute kidney injury, hepatic disease, alcohol toxicity, or heart failure. Let's go through the details:  Dehydration: While this patient has poor hygiene and could be neglecting fluid intake, his laboratory results don't support dehydration as a primary diagnosis. His sodium level is low, which would be unexpected in dehydration as it usually leads to hemoconcentration and elevated sodium levels.  Acute kidney injury (AKI): AKI can occur as a result of various factors such as severe dehydration, drug overdose, or other nephrotoxic insults. His elevated BUN and creatinine, along with the decreased urine output, strongly suggest renal impairment.  Hepatic disease: The patient has known hepatitis C, poor dentition, skin discoloration, and ascites (implied by abdominal distension with a positive fluid wave), suggesting cirrhosis or other significant hepatic disease. However, liver disease primarily impacts the kidneys through hepatorenal syndrome, which typically presents with very low urine sodium (<10 mEq/L), contrary to this patient's results.  Alcohol toxicity: Chronic alcohol misuse can lead to various complications, including liver disease and direct kidney damage. But alcohol-induced kidney damage doesn't usually cause such a severe decline in urine output.  Heart failure: This patient has a murmur and bibasilar crackles, which may suggest heart failure. Heart failure can cause renal impairment through low perfusion. However, heart failure often leads to hyponatremia and a low urine sodium concentration, not aligning with this patient's findings.  Given the patient's elevated BUN and creatinine, reduced urine output, and comparatively higher urine sodium level, his renal findings are most consistent with acute tubular necrosis (ATN). ATN often presents with renal impairment and an inability to concentrate urine, leading to wasted sodium (urine sodium >20 mEq/L). This condition is a common form of AKI and can be caused by a variety of insults, including ischemia (from hypotension, sepsis, or surgery) and nephrotoxic agents (medications, substances of abuse, or contrast dye). This patient's background of IV drug use and possible decreased perfusion from his hepatic disease may have contributed to the development of ATN.  **Model Answer**: ATN **Correct Answer**: Liver Failure (HRS)  **Incorrect Logic**: There are multiple incorrect logic statements in this rationale. First, dehydration can commonly lead to hyponatremia. Second, the patient’s production of 12 mmol of sodium over 24 hours is incorrectly classified by the model as an elevated urine sodium level when this value is more consistent with a low urine sodium. |

Supplementary Table 1.

Title: GPT-4 Incorrect Clinical Reasoning Examples

Description: Example GPT-4 MedQA questions where the model incorrectly answered the question and incorrect logic can be identified in the clinical reasoning rationale. Passages with incorrect logic are highlighted in yellow.
