# Supplementary material for: Diagnostic reasoning prompts reveal the potential for large language model interpretability in medicine
Source: NPJ Digit Med. 2024 Jan 24;7:20. doi: 10.1038/s41746-024-01010-1 (PMC10808088; doi:10.1038/s41746-024-01010-1)
Supplement: Supplementary file 8 — Supplementary Table 2 [file 41746_2024_1010_MOESM8_ESM.docx]

| **Question 1**  A 22-year-old man is brought to the emergency department by police after he was found undressing himself outside in 110F weather. The patient refuses to answer any questions as he suspects that his care team is spying on him. The patient recently started college and has presented to the emergency department once before for polysubstance intoxication. The patient attempts to assault a nurse, is restrained, and given diphenhydramine and haloperidol. When the patient is able to be examined, he is somnolent and non-responsive. His temperature is 104F (40C), blood pressure is 147/98 mmHg, pulse is 120/min, respirations are 17/min, and oxygen saturation is 98% on room air. He does not comply with any commands, and it is not possible to freely manipulate his extremities due to rigidity. His mucous membranes are dry. What is the most likely diagnosis?  **Rationale** (Differential Diagnosis):  The differential for this patient includes: heat stroke, neuroleptic malignant syndrome (NMS), serotonin syndrome, malignant hyperthermia, and anticholinergic toxicity. The patient has been given haloperidol, which is known to cause NMS. Symptoms of NMS include fever and muscle rigidity, which this patient displays. Further, the onset of symptoms in NMS can be very quick, especially when high potency neuroleptics like haloperidol are used. Although other syndromes like serotonin syndrome can cause similar symptoms, the patient's history does not indicate recent use of serotonergic drugs. Therefore, this patient most likely has neuroleptic malignant syndrome.  **Model Answer**: Neuroleptic Malignant Syndrome **Correct Answer:** Neuroleptic Malignant Syndrome |
| --- |
| **Question 2**  A 65-year-old man presents to his primary care physician for a change in his behavior over the past few months. Initially, the patient was noted to be behaving inappropriately including using foul language and grabbing people unexpectedly. This has progressed to a worsening of his memory and trouble caring for himself. His temperature is 98.1F (36.7C), blood pressure is 162/103 mmHg, pulse is 83/min, respirations are 13/min, and oxygen saturation is 98% on room air. Physical exam is notable for an elderly man who laughs inappropriately at times and who is a poor historian. When he is engaged in conversation, he exhibits word finding difficulty and is rather inattentive. What is the most likely diagnosis?  **Rationale** (Bayesian Inference):  The initial prior probability for frontotemporal dementia (FTD) in this case might be around 10% based on age and the general prevalence of the disease. However, considering the behavioral changes described, the probability can be increased. The patient's inappropriate behavior, including the use of foul language and unexpected physical contact, raises the probability to 30%. Frontotemporal dementia often initially manifests as changes in personality and behavior, which can seem out of character for the individual. His difficulty with word finding and attention further increases the probability to 50%. These are signs of language and cognitive impairment, which are common in FTD. The absence of other significant physical signs or symptoms (the patient's vital signs are mostly normal), coupled with the gradual progression over months, further increases the probability to 70%. These features are consistent with a neurodegenerative disorder like FTD. Therefore, based on the evidence provided, the most likely diagnosis is frontotemporal dementia.  **Model Answer:** Frontotemporal Dementia **Correct Answer:** Frontotemporal Dementia |
| **Question 3**  A 67-year-old woman presents from home hospice with a change in her mental status. She has seemed more confused lately and is unable to verbalize her symptoms. Her temperature is 102F (38.9C), blood pressure is 117/65 mmHg, pulse is 110/min, respirations are 19/min, and oxygen saturation is 95% on room air. Physical exam is notable for a right upper quadrant mass that elicits discomfort when palpated. Ultrasound is notable for pericholecystic fluid and gallbladder wall thickening without any gallstones. What is the most likely diagnosis?  **Rationale** (Intuitive Reasoning):  This patient presents with an altered mental status, fever, tachycardia, and a painful right upper quadrant mass with sonographic signs of pericholecystic fluid and gallbladder wall thickening. These findings suggest inflammation of the gallbladder. Given the absence of gallstones on ultrasound, this inflammation is likely secondary to acalculous cholecystitis, a condition that can occur in critically ill or immobile patients and is often associated with gallbladder stasis and bile concentration.  **Model Answer**: Acalculous Cholecystitis. **Correct Answer:** Acalculous Cholecysitis |
| **Question 4**  An 18-month-old girl is brought to the pediatrician by her mother for vaginal bleeding. The mother states that she noticed the bleeding today, which appeared as brown discharge with clots in the patient’s diaper. The mother denies frequent nosebleeds or easy bruising. She also denies any known trauma. She does mention that the patient has been limping and complaining of left leg pain since a fall 2 months ago. On physical exam, there are multiple 2-3 cm hyperpigmented patches on the patient’s trunk. There is bilateral enlargement of the breasts but no pubic hair. The vaginal orifice is normal and clear with an intact hymen. A plain radiograph of the left lower leg shows patchy areas of lytic bone and sclerosis within the femoral metaphysis. What is associated with the patient’s most likely diagnosis?  **Rationale** (Analytic Reasoning):  This patient's presentation of precocious puberty (bilateral breast enlargement), cafe-au-lait spots (hyperpigmented patches), and polyostotic fibrous dysplasia (lytic and sclerotic bone changes on the radiograph) is suggestive of McCune-Albright syndrome. This is a rare disorder caused by a postzygotic mutation in the GNAS1 gene, leading to mosaicism. The mutation causes constitutive activation of the Gs protein, leading to overproduction of cAMP and overactivity of downstream hormones such as estrogen, leading to precocious puberty. Furthermore, the mutation also affects bone-forming cells and results in polyostotic fibrous dysplasia as a result of abnormal bone remodeling.  **Model Answer:** McCune-Albright Syndrome. **Correct Answer**: McCune-Albright Syndrome |

Supplementary Table 2.

Title: GPT-4 Correct Clinical Reasoning Examples

Description: Example GPT-4 MedQA questions where the model correctly answered the question, providing a logical clinical reasoning rationale for its response.
